# Inferring an ancestral alginate lyase for improved stability and high-level expression using fed-batch fermentation

**DOI:** 10.3389/fmicb.2026.1799015

**Published:** 2026-03-17

**Authors:** Hongxiao Mu, Renquan Guo, Beichen Zhao, Zhuang Huang, Mingda Lei, Lei Lei, Jun Liu, Xing Wang, Zhenggang Xie

**Affiliations:** 1 School of Life Science and Technology, Wuhan Polytechnic University, Wuhan, China; 2 T&J Bio-Engineering (Shanghai) Co., Ltd., Shanghai, China; 3 Department of Chemical and Materials Engineering, The University of Auckland, Auckland, New Zealand; 4 Department of Biosciences, Faculty of Sciences, University Technology Malaysia, Johor, Malaysia

**Keywords:** alginate lyase, ancestral sequence inferring, fed-batch fermentation, heterologous expression, pH and thermal stability

## Abstract

Alginate lyases are valuable biocatalysts, but their industrial application is often constrained by limited stability and low production efficiency. In this study, an alginate lyase from *Flavobacterium* sp. was heterologously expressed in different host systems, among which *Escherichia coli* exhibited the highest enzymatic activity. Ancestral sequence inferring was subsequently applied to engineer improved variants, which were expressed and purified in *E. coli*. Two variants, AncAlyA1 and AncAlyA2, showed significantly enhanced catalytic activities, with increases of 132.4 and 87.3%, respectively, compared with the wild-type. Both variants also exhibited markedly improved thermal and pH stability. Notably, AncAlyA2 retained approximately 60% of its activity after incubation under extreme conditions, including 60 °C and pH 4.0 or 10.0. In addition, a fed-batch fermentation strategy based on combined glucose and yeast extract feeding was developed. Under optimized conditions, enzyme activities of 5164.8 U/mL and 4220.1 U/mL were achieved for AncAlyA1 and AncAlyA2, respectively. This study provides a promising strategy for industrial-scale production of alginate lyase.

## 1 Introduction

Alginate, an acidic linear polysaccharide, consists of *β*-D-mannuronic acid (M) and *α*-L-guluronic acid (G) residues linked by 1,4-glycosidic bonds, and arranged in different types including homopolymeric M- or G-blocks or as heteropolymeric MG sequences (Gimpel et al., 2018). As a naturally derived polymer extracted from brown algae, alginate exhibits excellent biocompatibility and environmental friendliness. It is widely used in the food industry as a thickener and stabilizer and has also been extensively applied in biomedical fields, such as wound dressings and drug delivery systems, in the form of hydrogels (Dhandayuthapani et al., 2011; Aderibigbe and Buyana, 2018; Reddy et al., 2018). However, the broader application of sodium alginate is limited by several inherent drawbacks, including high molecular weight, limited water solubility, and low bioavailability. In contrast, alginate-derived oligosaccharides (AOS), which are degradation products of sodium alginate, effectively overcome the limitations associated with polysaccharides, such as large molecular size and poor solubility, and exhibit diverse biological activities, including antioxidant activity, immunomodulatory effects, and regulation of blood glucose and lipid metabolism (Hu et al., 2004; Iwamoto et al., 2005).

Alginate lyases catalyze the degradation of alginate via a *β*-elimination mechanism, generating unsaturated uronic acids characterized by a strong absorbance at 235 nm (Dong et al., 2014). These enzymes have been identified in a wide range of organisms, including marine bacteria, fungi, algae, mollusks, as well as terrestrial soil bacteria, nitrogen-fixing bacteria, and *Pseudomonas* phages (Zhang et al., 2004; Pilgaard et al., 2019; Peng et al., 2018). According to the Carbohydrate-Active Enzymes (CAZy) database, alginate lyases are classified into 14 kinds of polysaccharide lyase (PL) families, among which PL5 and PL7 are the most prevalent (Peng et al., 2018; Jouanneau et al., 2021). Alginate lyases from natural sources often exhibit limitations such as low specific activity, poor stability, and difficulties in purification, which restrict their industrial application for large-scale AOS production. Consequently, improving the catalytic performance of alginate lyases through rational or semi-rational protein engineering has become a major research focus. Current strategies, including site-directed mutagenesis (Jang et al., 2016; Wang et al., 2018; Lyu et al., 2019; Qin et al., 2018; Tang et al., 2020; Zhang et al., 2023; Yuan et al., 2025), domain truncation (Hu et al., 2019a; Yan et al., 2019; Fu et al., 2024; Grobler et al., 2024), and recombination (Zhang et al., 2019; Yang et al., 2019; Hu et al., 2021; Lyu et al., 2018; Hu et al., 2019b), have been successfully employed to enhance catalytic efficiency, thermostability, and product specificity. Nevertheless, the extent of improvement achieved so far remains insufficient to meet the stringent requirements of industrial-scale AOS production, highlighting the need for further research efforts.

In addition, natural alginate lyases are often derived from non-platform microbial strains, which typically exhibit low expression levels, thereby limiting their industrial applicability. To fully exploit the diverse catalytic properties of alginate lyases from different species, platform strains can be employed as heterologous expression hosts. Optimization of host characteristics can significantly enhance alginate lyase production, consequently increasing the yields of alginate oligosaccharides (AOS) and unsaturated oligosaccharides (DEH). For example, *Escherichia coli* has been widely recognized in several studies as a suitable host for enhancing the expression of alginate lyases, owing to its rapid growth rate, low fermentation cost, and high protein production efficiency (Grobler et al., 2024; Falak et al., 2022; Wang et al., 2024, 2025). Similarly, *Bacillus subtilis* and *Komagataella phaffii* are widely used in industrial applications due to their strong secretory capacity for heterologous proteins, ease of genetic manipulation, and well-established genetic systems (Zheng et al., 2023; Rhein-Knudsen et al., 2021; Zhou et al., 2024; Li et al., 2018). Nevertheless, current studies predominantly focus on heterologous expression and enzymatic characterization of alginate lyases, with fermentation investigations largely confined to shake-flask cultures. There remains a notable lack of studies aimed at optimizing fermentation processes at the bioreactor scale to enhance enzyme production.

In this study, an alginate lyase derived from *Flavobacterium* sp. was investigated for heterologous expression in three platform microbial hosts: *E. coli*, *Bacillus licheniformis*, and *K. phaffii*, and their expression efficiencies were comparatively evaluated. Subsequently, ancestral sequence reconstruction (ASR) was performed by constructing a phylogenetic tree and applying multiple evolutionary models to infer ancestral amino acid sequences from a curated set of homologous proteins. Recombinant strains using the optimal host were constructed to express either the wild-type enzyme or reconstructed ancestral variants, and their catalytic properties were systematically compared. Finally, fed-batch fermentation of each recombinant strain was carried out in a 5-L bioreactor, with optimization of key operational parameters related to induction and nutrient feeding strategies. The overarching aim of this study is to develop a robust recombinant strain capable of high-level production of a thermostable alginate lyase under scalable fermentation conditions, thereby providing a technical foundation for its industrial application.

## 2 Materials and methods

### 2.1 Strains, plasmids and culture media

When *E. coli* BL21 (DE3) was used as the expression host, the expression vector pET21a(+) was employed. For heterologous expression in *K. phaffii* X-33, the vector pPIC9K was utilized. In the case of *Bacillus licheniformis* BL11, the expression vector pHY300PLK was used, which harbors the *B. subtilis* 168 promoter P_43_, the saccharification enzyme signal peptide SP_SacC_, and the *B. licheniformis* amylase terminator T_amyL_. *Escherichia coli* DH5*α* served as the host for construction of each plasmid.

Seed cultures of *E. coli* and *B. licheniformis* were cultivated in LB medium at 37 °C for 12 h with shaking at 230 r/min. Seed cultures of *K. phaffii* were grown in YPD medium at 28 °C for 24 h under the same agitation conditions. The culture media used for shake-flask and fermenter culture of *E. coli* were prepared according to a previously reported method (Song et al., 2013). The fermentation medium for bioreactor cultivation is explicitly defined as: glucose 10 g/L, yeast extract 4 g/L, KH₂PO₄ 13.5 g/L, (NH₄)₂HPO₄ 4 g/L, citric acid monohydrate 2 g/L and MgSO₄ 1 g/L adjusted to pH 7.0. The trace element solution composition is: FeSO₄·7H₂O 10 g/L, CuSO₄·5H₂O 3 g/L, MnSO₄·H₂O 0.2 g/L, ZnSO₄·7H₂O 5.25 g/L, (NH₄)Mo₇O₂₄ 0.1 g/L, Na₂B₄O₇·10H₂O 0.2 g/L and CaCl₂ 2 g/L dissolved in 1 mol/L HCl and sterilized by filtration. It is added to the fermentation medium at 10 mL/L. The glucose feed solution concentration is 500 g/L and the yeast extract feed solution concentration is 100 g/L. The medium used for shake-flask fermentation of *B. licheniformis* was based on our previous study (Li et al., 2023), while the fermentation medium for *K. phaffii* was prepared following the protocol described in the literature (Li et al., 2018). Based on necessity, an appropriate antibiotic was added to broth at the following final concentrations: 100 μg/mL of ampicillin for *E. coli*, 20 μg/mL of tetracycline for *B. licheniformis*, and 250 μg/mL of G418 for *K. phaffii*.

### 2.2 Cloning, expression, and purification

All expression vectors were constructed using the same strategy. Target genes were ligated into the corresponding expression vectors using the ClonExpress II One Step Cloning Kit (Vazyme, Nanjing, China) according to the manufacturer’s instruction. The primers used for plasmid construction are listed in Supplementary Table S1. The ligation products were transformed into *E. coli* DH5α competent cells via the calcium chloride method. Positive transformants were screened by colony PCR using the corresponding verification primers (Supplementary Table S1), and the PCR products were subsequently sequenced by Sangon Biotech (Shanghai, China).

Plasmids confirmed to be correct were extracted using the Plasmid Mini Kit (Beijing Solarbio Science & Technology Co., Ltd., Beijing, China) and introduced into the appropriate host strains by electroporation. Electroporation was performed using 0.2 cm gap cuvettes under the following conditions: 1.8 kV for *E. coli* BL21 (DE3), 2.2 kV for *B. licheniformis*, and 2.0 kV for *K. phaffii*. Detailed electroporation procedures were carried out as previously described (Du et al., 2021; Wang et al., 2020; Mohammadzadeh et al., 2021).

The crude enzyme supernatant obtained from disruption of *E. coli* by high-pressure homogenization, was collected and subjected to protein purification using the Ni^2+^ chelating resin column. Next, the eluted protein solution was subsequently dialyzed three times to remove imidazole and residual metal ions. After dialysis, the protein concentration and purity were determined by the bicinchoninic acid (BCA) assay and sodium dodecyl sulfate–polyacrylamide gel electrophoresis (SDS-PAGE), respectively. SDS-PAGE analysis was performed using 12% polyacrylamide gels. For both flask cultivation and bioreactor fermentation samples, 10 μL of undiluted sample was loaded per lane. Prior to electrophoresis, all samples underwent standardized pretreatment: fermentation supernatants were centrifuged at 12,000–16,000 g for 10–15 min at 4 °C to remove cellular debris and white precipitates. The clear supernatant was then mixed with 5 × Loading Buffer in a 4:1 ratio, heated at 100 °C for 10 min for protein denaturation, and briefly centrifuged (1 min) to pellet debris before loading.

### 2.3 Ancestral sequence reconstruction

Ancestral sequence reconstruction was carried out based on the protocol described in reference (Lei et al., 2023), with the detailed procedures outlined as follows: Sequences of alginate lyase family members were retrieved from the National Center for Biotechnology Information (NCBI) non-redundant protein database (nr) using the BLASTp tool with default parameters. Sequences exhibiting ≥95% query coverage were retained for further analysis. Redundant sequences sharing ≥90% identity were removed using CD-HIT (version 4.8.1). Multiple sequence alignment was then performed using MUSCLE (Edgar, 2004), and gap-rich regions were trimmed using trimAl (version 1.2) (Capella-Gutiérrez et al., 2009). Phylogenetic analysis was conducted using IQ-TREE (version 1.6.12) to select the optimal evolutionary model and construct a maximum-likelihood tree. The best-fitting substitution model determined by IQ-TREE was LG + F + I + G4 (Nguyen et al., 2015). The reliability of internal nodes was evaluated using bootstrap analysis with 10,000 replicates. Ancestral sequence reconstruction was subsequently performed using the FastML algorithm (version 3.11) based on the generated alignment and phylogenetic tree (Ashkenazy et al., 2012). The LG substitution model with a gamma-distributed rate variation was applied during the reconstruction process.

### 2.4 Enzyme activity and biochemical characterization assay

The enzyme activity of alginate lyase was determined by the 3,5-dinitrosalicylic acid (DNS) method (Chen et al., 2025). Briefly, 0.2 mL of diluted enzyme solution was mixed with 0.8 mL of 0.7% (w/v) sodium alginate solution (dissolved in Phosphate buffered saline) and incubated at 37 °C for 30 min. Then, 2 mL of DNS reagent was added into the mixture, and the solution was incubated in a boiling water bath for 5 min, cooled to room temperature, and diluted to 12 mL with distilled water. A blank control was prepared using a heat-inactivated enzyme under identical conditions. Absorbance was measured at 540 nm with three replicates per reaction. One unit of enzyme activity (U) was defined as the amount of enzyme required to release 1 μg of reducing sugar per minute. Protein concentration was determined using a bicinchoninic acid protein assay kit (Beijing Solarbio Science & Technology Co., Ltd., Beijing, China). Specific activity (U/mg) was calculated as the ratio of enzyme activity (U/mL) to protein concentration (mg/mL).

The optimal pH of the enzyme was determined by adjusting the pH of the sodium alginate substrate solution prepared in phosphate-buffered saline (PBS) using 6 M NaOH or phosphoric acid. Enzyme reactions were carried out at pH values ranging from 3.0–10.0 for 30 min, after which the enzymatic activity was measured. The pH stability of the enzyme was evaluated by adjusting the pH of the diluted enzyme solution with the same acid–base system. The enzyme solutions were incubated at pH values ranging from 4.0 to 10.0 at 37 °C for 30 min, followed by determination of the residual enzymatic activity under standard assay conditions. The optimal temperature of the enzyme was determined by incubating the enzyme–substrate mixture at temperatures ranging from 10 to 70 °C for 30 min. Thermal stability was evaluated by incubating the enzyme at different temperatures ranging from 45 to 70 °C for 30 min, the residual enzymatic activity was then determined under standard reaction conditions.

### 2.5 Fed-batch cultures in 5 L stirred fermenters and analysis methods

Fed-batch fermentation was performed in a 5-L mechanically stirred bioreactor. The initial fermentation temperature was maintained at 37 °C and subsequently reduced to 28 °C upon induction. Throughout the fermentation process, the pH was maintained at 7.0 by automatic addition of 6 M aqueous ammonia. The initial agitation speed and aeration rate were set at 300 r/min and 1.0 vvm, respectively. As dissolved oxygen (DO) decreased during cultivation, both agitation speed and aeration rate were gradually increased to their maximum values of 700 r/min and 2.0 vvm, which were then maintained constant. The initial working volume was 2.5 L, and the inoculation volume was 3% (v/v). When the initial carbon source was completely consumed, glucose feeding and yeast extract feeding were initiated simultaneously. Induction was carried out by adding IPTG when the biomass reached a predetermined level, and the induction phase was maintained for 20 h.

Three glucose feeding strategies were evaluated in this study: 5 and 10 g/L/h constant-rate feeding, and DO-stat–controlled glucose feeding. In addition, three yeast extract feeding rates (0, 0.4, and 1.0 g/L/h) were investigated. The effects of induction at different biomass levels (OD₆₀₀ = 30, 60, and 90) and different IPTG concentrations (0.1, 0.2, and 0.4 mM) were also systematically evaluated.

Biomass was measured through the determination of the optical density at 600 nm. Glucose concentration was determined using an SBA-40C bioanalyzer (Academy of Sciences, Ji’nan, China) (Cai et al., 2018). All samples were analyzed in triplicate, with the results reported as the mean ± the standard deviation for each data point.

## 3 Results

### 3.1 Over-expression of alginate lyase in different expression systems

**Figure 1:**
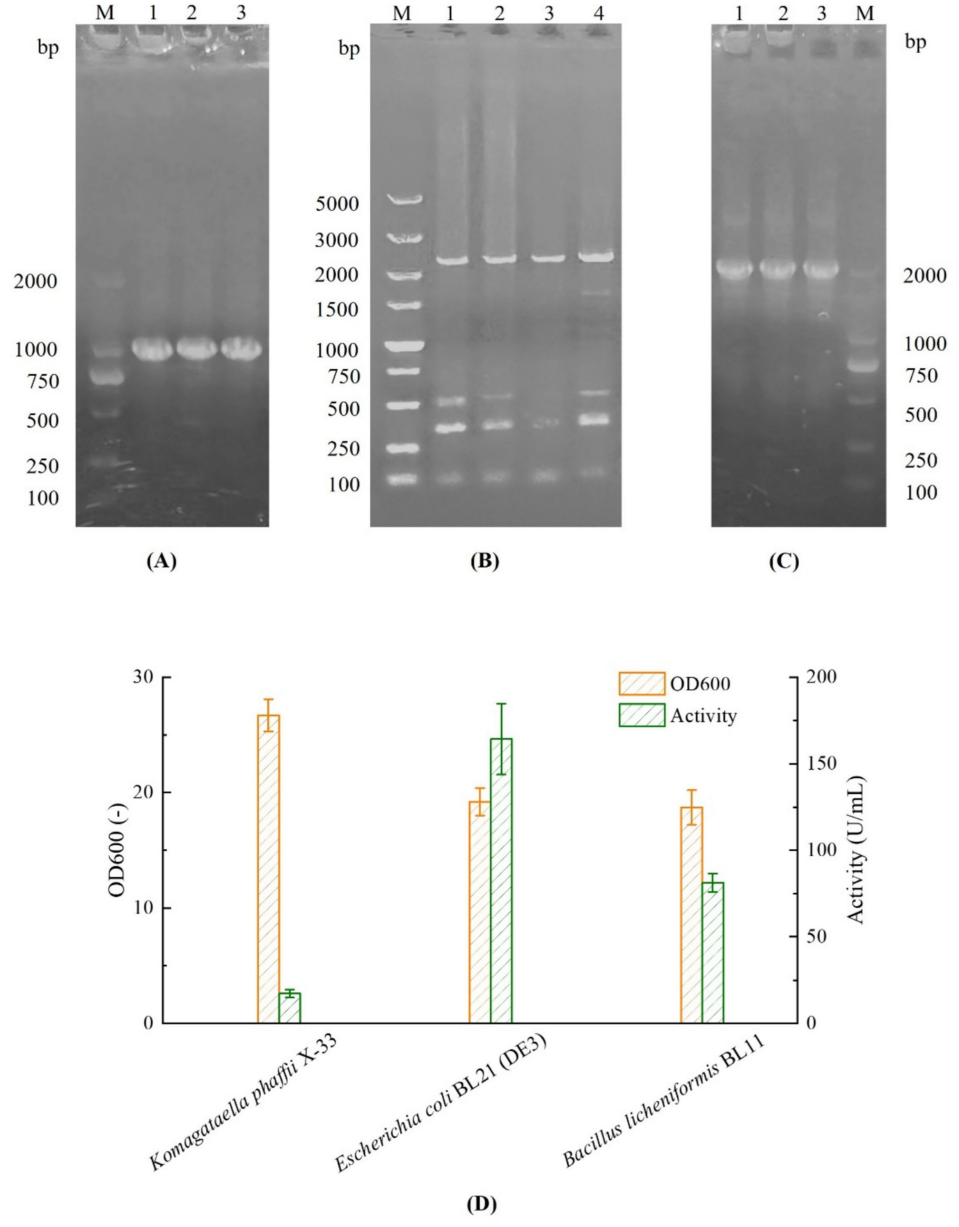
PCR verification and fermentation profiles of different recombinants in shake-flask. **(A)** PCR products of *E. coli* BL21-FlAlyA. Lanes 1–3: PCR products of BL21-FlAlyA (984 bp), lane M: DL2000 DNA marker; **(B)** PCR products of *K. phaffii* X33-FlAlyA. Lanes 1–4: PCR products of X33-FlAlyA (2,421 bp), lane M: DL5000 DNA marker; **(C)** PCR products of *B. licheniformis* BL11-FlAlyA. Lanes 1–3: PCR products of BL11-FlAlyA (2028 bp), lane M: DL2000 DNA marker; **(D)** Fermentation profiles comparison between different recombinants.

The gene *FlAlyA* (GenBank ID: AB898059) encodes an alginate lyase from *Flavobacterium* sp. and consists of 260 amino acid residues with a theoretical molecular weight of approximately 30 kDa (Inoue et al., 2014). The *FlAlyA* gene sequence was codon-optimized for three heterologous expression hosts, including *E. coli*, *B. licheniformis*, and *K. phaffii*. The optimized sequences were subsequently cloned into the corresponding expression vectors. PCR verification confirmed that the recombinant plasmids were successfully introduced into the respective host strains, as shown in Figures 1A–C.

Subsequently, each recombinant strain was cultivated in shake-flask fermentation, and the results are shown in Figure 1D. *Komagataella phaffii* exhibited the highest biomass, reaching an OD₆₀₀ value of 26.7; however, the extracellular enzyme activity in the culture supernatant was only 17.2 U/mL, which was the lowest among all tested strains. Similarly, *B. licheniformis* exhibited secretory expression, achieving a final OD₆₀₀ of 18.7 and an extracellular enzyme activity of 81.2 U/mL. In contrast, *E. coli* expressed the enzyme intracellularly and reached an OD₆₀₀ of 19.2, while the enzyme activity in the cell lysate reached 164.2 U/mL, representing the highest activity among the three hosts. This activity was approximately 9.55-fold and 2.02-fold higher than those obtained with *K. phaffii* and *B. licheniformis*, respectively. Therefore, *E. coli* BL21(DE3) was selected as the expression host for subsequent protein engineering studies.

### 3.2 Design and generation of infer ancestral mutants

**Figure 2:**
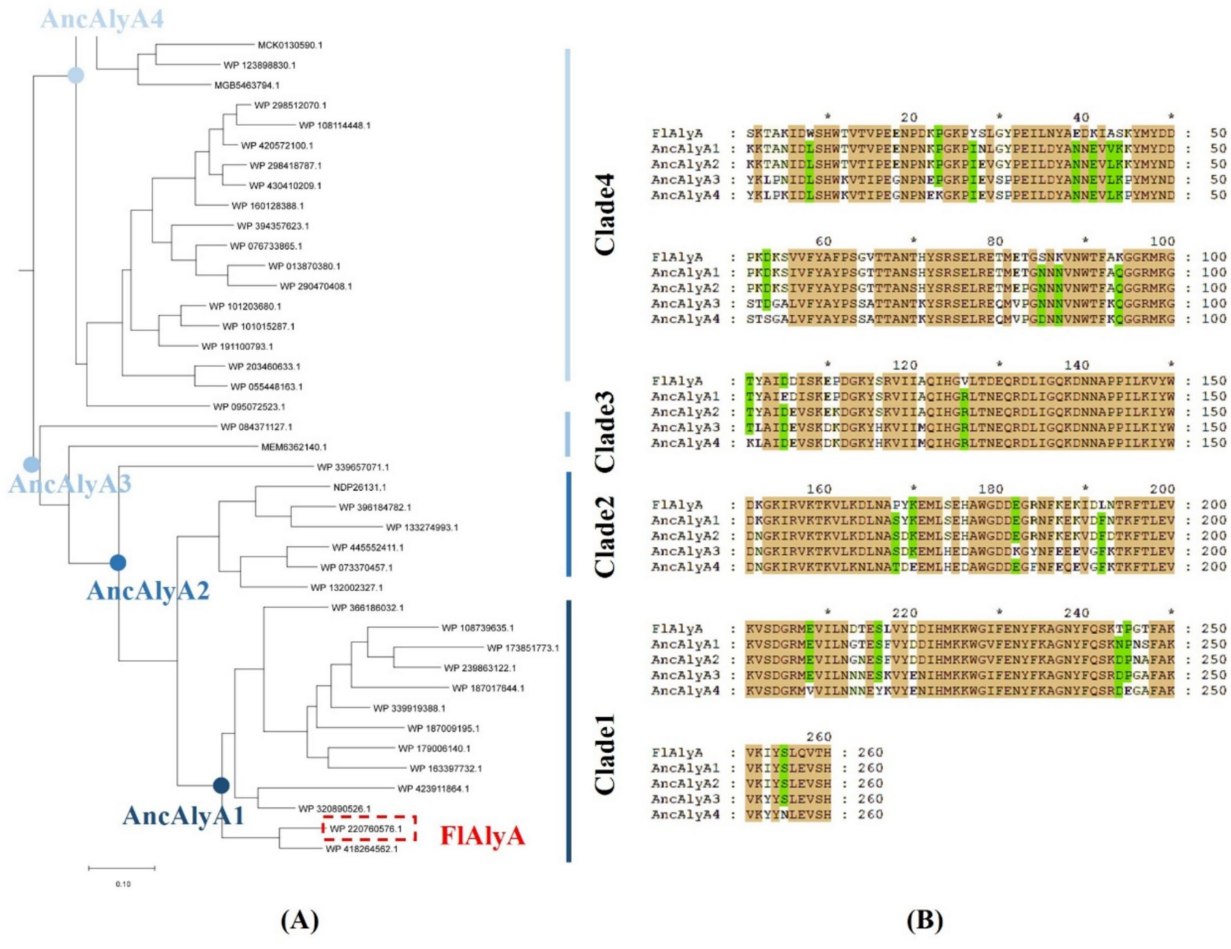
Phylogenetic tree of FlAlyA and alignment of the inferred ancestral sequences. **(A)** Maximum-likelihood phylogenetic tree of FlAlyA and its optimized variants (AncAlyA1-4). Branches are colored in black to indicate evolutionary pathways. Ancestral nodes are marked with blue dots (left to right: AncAlyA4 → AncAlyA3 → AncAlyA2 → AncAlyA1), culminating in the modern FlAlyA sequence (red dashed border). The tree topology reveals clear evolutionary stages with AncAlyA1-4 representing sequential ancestors leading to FlAlyA. **(B)** Sequence alignment of FlAlyA and its ancestors. Residues differing from FlAlyA are highlighted with colored blocks following ClustalX coloring scheme. Consensus residues are denoted by dots.

The wild-type alginate lyase from *Flavobacterium* sp. (FlAlyA) was used as a query to search the NCBI protein database (Accessed August 22, 2024) in order to infer ancestral alginate lyase variants, and all the NCBI accession numbers of the alginate lyase family sequences were listed in Figure 2A. Homologous sequences with sequence identities greater than 55% were retrieved and subsequently dereplicated using CD-HIT with a 95% sequence identity cutoff. The remaining sequences were aligned using MUSCLE (Edgar, 2004), followed by gap trimming with trimAI (Capella-Gutiérrez et al., 2009). Based on the resulting alignment, a maximum-likelihood phylogenetic tree was constructed, in which FlAlyA-related sequences were clustered into four major clades (Figure 2A). In this study, four ancestral nodes were reconstructed to represent the evolutionary divergence of alginate lyases. Ancestral sequence reconstruction was performed using the maximum-likelihood algorithm implemented in FastML (Ashkenazy et al., 2012). The inferred ancestral alginate lyases were designated as AncAlyA variants. Among them, AncAlyA1 represents the ancestral sequence of clade 1 and shares approximately 90% sequence identity with the extant FlAlyA, whereas AncAlyA4 was inferred as the last common ancestor of all sequences included in the phylogenetic analysis. Sequence divergence analysis (Figure 2B) indicated that AncAlyA1 differed from FlAlyA by 38 amino acid residues, whereas AncAlyA4 exhibited 86 amino acid substitutions relative to the wild-type enzyme.

**Figure 3:**
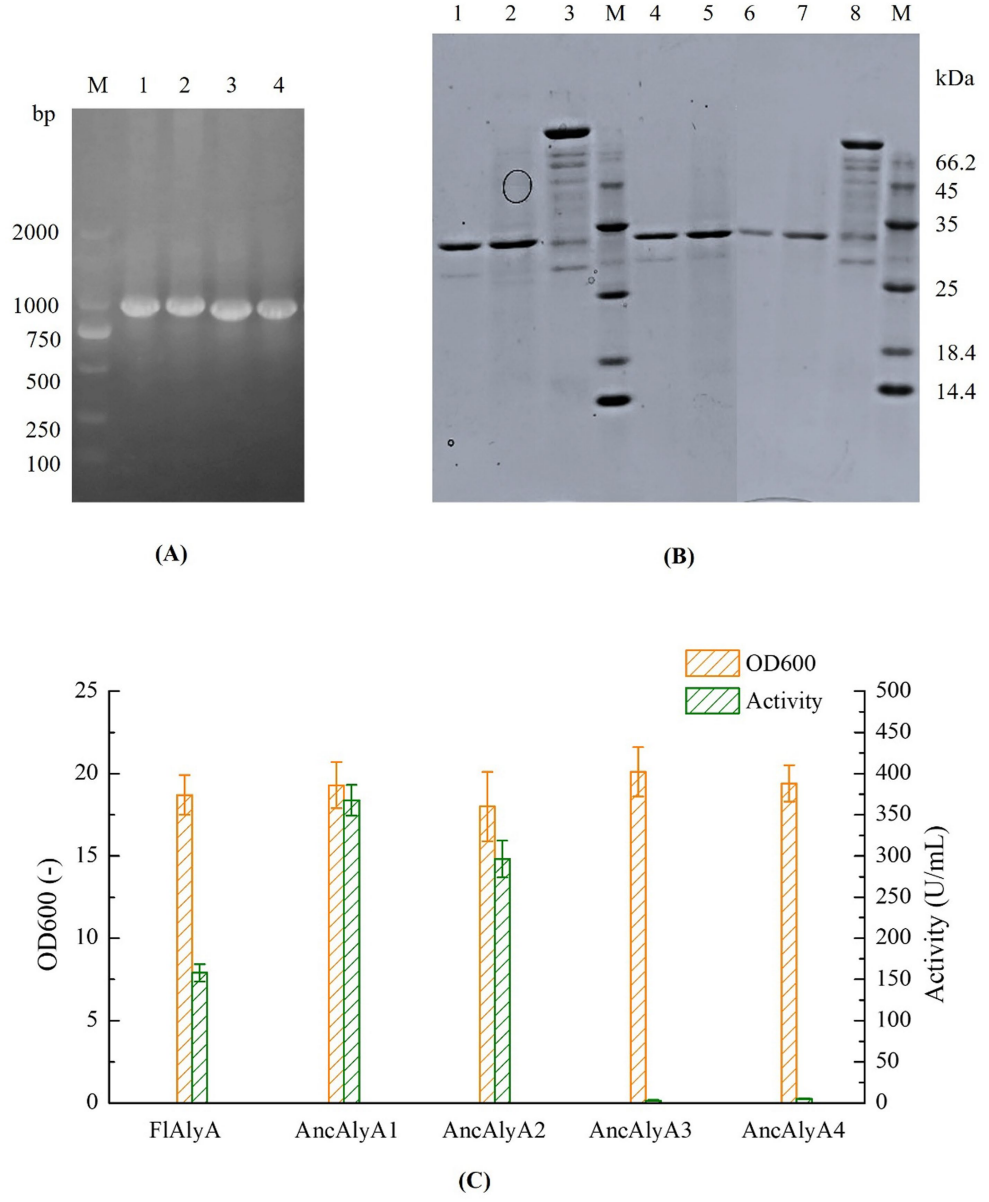
PCR verification, SDS-PAGE analysis, and shake-flask fermentation of *E. coli* BL21-FlAlyA and its mutants. **(A)** PCR products of four ancestral variants. Lanes 1–4: AncAlyA1-4 (984 bp), Lane M: DL2000 DNA marker; **(B)** SDS-PAGE analysis for the cell lysate supernatants and purified proteins. Lane 1: purified FlAlyA (30 kDa); Line 2: crude FlAlyA; Line 3 and Line 8: 1 g/L bovine serum albumin (BSA); Line 4: purified AncAlyA1 (30 kDa); Line 5: crude AncAlyA1; Line 6: purified AncAlyA2 (30 kDa); Line 7: crude AncAlyA2; Line M: protein marker; **(C)** Fermentation profiles comparison between *E. coli* BL21-FlAlyA and its mutants.

To evaluate the activities of engineered alginate lyases, synthetic genes encoding four ancestral variants were codon-optimized for expression in *E. coli* and heterologously expressed using the pET-21a/*E. coli* BL21(DE3) system. Recombinant strains harboring each ancestral sequence were successfully screened and verified by PCR, as shown in Figure 3A. Subsequently, shake-flask fermentation was conducted for both the ancestral variants and the wild-type FlAlyA, followed by cell disruption to obtain cell lysate supernatants. SDS-PAGE analysis revealed that all four ancestral variants exhibited a distinct protein band with an apparent molecular weight of approximately 30 kDa (Figure 3B; Supplementary Figure S1), confirming successful expression of the engineered alginate lyases. In addition, the biomass of the recombinant strains was comparable to that of the wild-type strain (Figure 3C). However, enzyme activity assays demonstrated that the cell lysate supernatants of AncAlyA3 and AncAlyA4 exhibited no detectable alginate lyase activity. In contrast, AncAlyA1 and AncAlyA2 displayed enzymatic activities of 367.5 U/mL and 296.2 U/mL, respectively, corresponding to increases of approximately 132.4 and 87.3% relative to the wild-type FlAlyA. SDS-PAGE analysis (Figure 3B) demonstrates that the expression levels of AncAlyA1 and AncAlyA2 are comparable to that of wild-type FlAlyA. This comparable expression suggests that AncAlyA1 and AncAlyA2 exhibit higher specific activity (U/mg-protein) than wild-type FlAlyA, a point to be detailed in the subsequent biochemical characterization section. It is important to note that minor enzymatic losses and operational variations during sample dilution, cell disruption, purification, and activity assays may lead to discrepancies between the magnitudes of specific activity increases and volumetric activity (U/mL) increases. However, the consistent trend of enhanced activity is maintained across these analyses.

### 3.3 Biochemical characterization of FlAlyA and its ancestral variants

**Figure 4:**
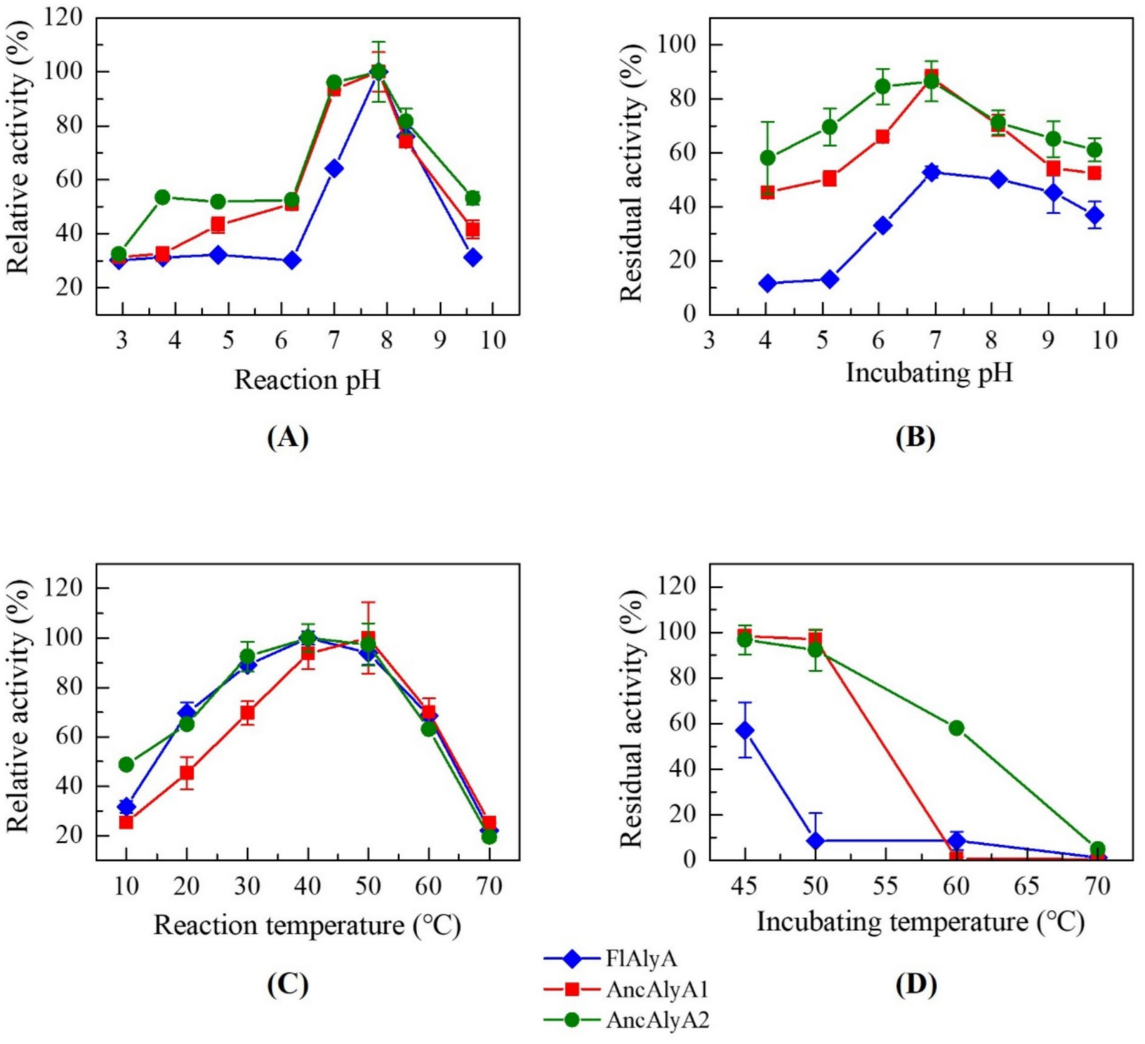
Biochemical characterization of FlAlyA and its ancestral variants. **(A)** Optimal reaction pH; **(B)** pH stability; **(C)** Optimal reaction temperature; **(D)** Temperature stability.

The wild-type FlAlyA and the ancestral variants AncAlyA1 and AncAlyA2 were purified using a C-terminal His-tag, and the purity of the proteins was confirmed by SDS-PAGE analysis (Figure 3B). Due to their no detectable catalytic activity, AncAlyA3 and AncAlyA4 were not subjected to further characterization. Subsequently, the effects of pH on enzyme activity and stability were investigated for both the ancestral variants and the wild-type enzyme. As shown in Figure 4A, FlAlyA and the two ancestral variants exhibited an optimal pH of 7.8. Under acidic (pH < 6.0) or alkaline (pH > 9.0) conditions, the relative activity of FlAlyA decreased sharply to approximately 30%, whereas the ancestral variants maintained higher activity levels, generally exceeding 50%. Furthermore, FlAlyA retained less than 15% of its initial activity after 30 min of incubation at pH 4.0–5.0 and approximately 40% activity at pH 9.0–10.0. In contrast, AncAlyA1 and AncAlyA2 retained around 50% of their activity under both acidic and alkaline conditions (Figure 4B). These results indicate that the ancestral variants exhibit enhanced pH tolerance and maintain higher catalytic efficiency over a broader pH range compared with the wild-type enzyme.

The optimal reaction temperature for FlAlyA and AncAlyA2 was determined to be 40 °C, whereas AncAlyA1 exhibited an optimal temperature of 50 °C (Figure 4C). Overall, the enzymatic activities of FlAlyA and its ancestral variants showed only minor variation across the tested temperature range. However, pronounced differences were observed in their thermal stability profiles. After incubation at 45 °C for 0.5 h, AncAlyA1 and AncAlyA2 retained 98.5 and 96.6% of their initial activities, respectively, whereas FlAlyA retained only 57.1% activity (Figure 4D). Furthermore, following incubation at 50 °C, FlAlyA exhibited almost no residual activity, while both ancestral variants retained more than 90% activity. Notably, AncAlyA2 still maintained 58.0% activity after incubation at 60 °C for 0.5 h. These findings indicate that the ancestral variants possess markedly enhanced thermal stability, with AncAlyA2 showing the most pronounced improvement.

The kinetic parameters of wild-type FlAlyA and its ancestral variants were determined under their respective optimal conditions, and the results are summarized in Table 1. AncAlyA1 and AncAlyA2 exhibited *V_max_* values of 1117.56 and 1225.34 μmol/min, corresponding to increases of 15.1 and 26.1%, respectively, compared with FlAlyA. In addition, their catalytic efficiencies (*k_cat_/K_m_*) reached 229.02 and 204.91, representing enhancements of 66.9 and 49.4% relative to the wild-type enzyme. These results demonstrate that ancestral reconstruction significantly improved both the catalytic activity and efficiency of alginate lyase.

**Table 1:** Kinetic parameters of FlAlyA and its two ancestral variants.

Protein	Specific activity (U/mg)	$V_{\max}$ (μmol/mg/min)	$k_{\mathrm{cat}}$ (s^−1^)	$K_{m}\;$ (mg/mL)	kcatKm (mL/mg/s)
FlAlyA	746.30	971.34	485.7	3.54	137.20
AncAlyA1	1,064.77	1,117.56	558.8	2.44	229.02
AncAlyA2	958.55	1,225.34	612.67	2.99	204.91

### 3.4 Improving alginate lyase production by fed-batch fermentation with an optimal feeding strategy of glucose and yeast extract

In this study, fed-batch fermentation was performed in a 5-L bioreactor using a recombinant strain expressing AncAlyA1 as the production host. Key process parameters, including glucose feeding strategy, yeast extract supplementation rate, induction biomass, and IPTG concentration, were systematically optimized, and the results are summarized in the corresponding figure.

Initially, three glucose feeding strategies were evaluated: constant feeding at rates of 10 g/L/h and 5 g/L/h, and DO-STAT feedback-controlled feeding, and the results show in Supplementary Figure S2. Constant feeding at 10 g/L/h resulted in the highest specific growth rate, reaching an OD₆₀₀ of 159.2 at 21 h. However, residual glucose began to accumulate after 15 h and reached 53 g/L by the end of fermentation. Under this condition, enzyme activity peaked at 2788.3 U/mL at 18 h and subsequently plateaued, resulting in the lowest final activity (2918.9 U/mL) among the three strategies. In contrast, DO-STAT-controlled feeding maintained low residual glucose levels while supporting robust cell growth, yielding a final enzyme activity of 3737.4 U/mL. Constant feeding at 5 g/L/h led to the lowest growth rate but still achieved a high biomass (OD₆₀₀ = 154.8), with negligible glucose accumulation. Notably, this condition produced the highest enzyme activity of 4983.7 U/mL. These results indicate that excessive glucose supply inhibits enzyme production, whereas a moderate feeding rate of 5 g/L/h favors both cell growth and enzyme synthesis.

Subsequently, yeast extract was supplied at constant rates of 0, 0.4, and 1.0 g/L/h. Although the highest biomass (OD₆₀₀ = 189.8) was obtained at 1.0 g/L/h, this condition resulted in the lowest enzyme activity (Supplementary Figure S3). Supplementation at 0.4 g/L/h led to slightly improved biomass and enzyme production compared with the non-supplemented group. Notably, when induction began at OD₆₀₀ values of 30, 60, and 90, the corresponding growth durations were 10 h, 12 h, and 15 h, respectively. To ensure uniform protein expression time across conditions, the induction period was consistently maintained at 20 h for all samples, as shown in Supplementary Figure S4. Induction at OD₆₀₀ values of 30 and 60 resulted in high enzyme activities (>4,500 U/mL), whereas induction at OD₆₀₀ 90 prolonged fermentation time and significantly reduced enzyme yield (3217.8 U/mL). Furthermore, varying IPTG concentrations (0.1, 0.2, and 0.4 mM) had no significant effect on cell growth, glucose consumption, or enzyme activity (Supplementary Figure S5). Considering economic feasibility, 0.1 mM IPTG was selected for subsequent experiments.

**Figure 5:**
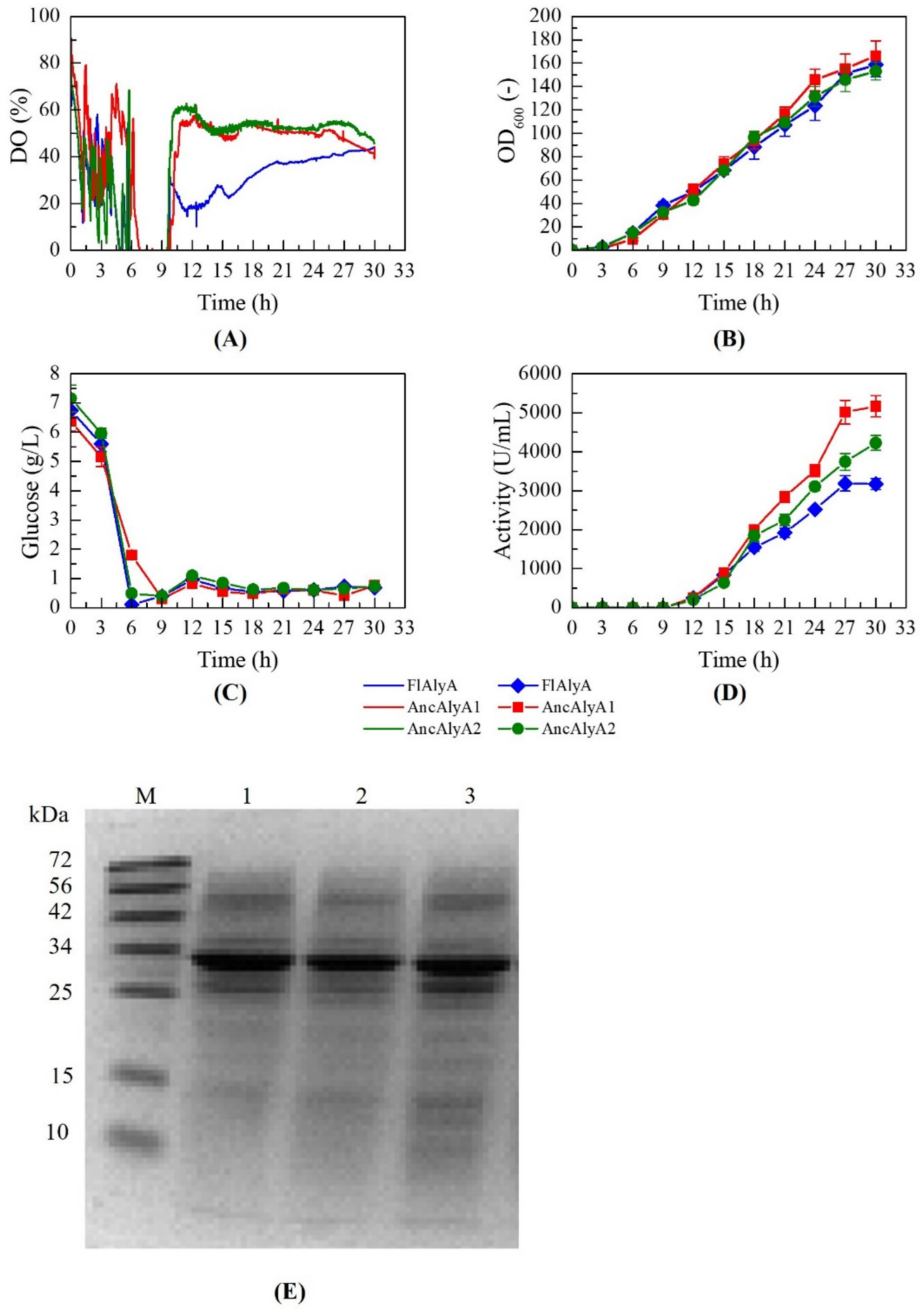
Comparative analysis of fed-batch fermentation profiles between recombinants expressing FlAlyA and its ancestral variants. **(A)** DO; **(B)** Biomass (OD_600_); **(C)** Residual glucose; **(D)** Enzyme activity; **(E)** SDS-PAGE analysis of alginate lyase production by different recombinants, Line 1: FlAlyA; Line 2: AncAlyA1; Line 3: AncAlyA2; Line M: protein marker.

Based on these optimization results, fed-batch fermentations of recombinant strains expressing FlAlyA, AncAlyA1, and AncAlyA2 were conducted under the following conditions: glucose feeding at 5 g/L/h, yeast extract supplementation at 0.4 g/L/h, and induction with 0.1 mM IPTG at an OD₆₀₀ of 30. As shown in Figure 5, all strains exhibited comparable growth and glucose consumption profiles. The wild-type FlAlyA exhibited an activity of 3170.5 U/mL under optimized fermentation conditions, while the final enzyme activities reached 5164.8 U/mL for AncAlyA1 and 4220.1 U/mL for AncAlyA2, representing increases of 62.9 and 33.1%, respectively, compared with the wild-type FlAlyA.

## 4 Discussion

Previous studies have demonstrated that naturally derived alginate lyases generally exhibit limited thermal and pH stability. Enzyme activity decreases markedly when the temperature exceeds 40 °C or when the pH deviates toward acidic (<6.5) or alkaline (>9.0) conditions (Inoue et al., 2014; Meng et al., 2021). These intrinsic limitations severely restrict their applicability in industrial processes. Therefore, rational enzyme engineering based on a comprehensive understanding of enzyme origin, structure, and function has become a major research focus for improving the catalytic performance of alginate lyases. Ancestral sequence reconstruction (ASR) has emerged as a promising alternative strategy to conventional directed evolution. While directed evolution mimics natural selection through random mutagenesis and high-throughput screening, ASR applies a computational retrospective approach to infer ancestral protein sequences based on evolutionary relationships. Previous studies have demonstrated that ancestral variants often exhibit superior catalytic activity, enhanced thermal stability, and broader pH tolerance compared with their modern counterparts (Lei et al., 2023; Zhang et al., 2024; Babkova et al., 2017). In the present study, four ancestral variants of alginate lyase were reconstructed using ASR. Among them, recombinant strains expressing AncAlyA1 and AncAlyA2 exhibited substantially enhanced enzymatic activities of 367.5 U/mL and 296.2 U/mL, respectively, corresponding to increases of 132.4 and 87.3% relative to the wild-type FlAlyA. Moreover, both variants displayed significantly improved pH and thermal stability. Notably, AncAlyA2 retained approximately 60% of its residual activity after 30 min of incubation under extreme conditions, including acidic (pH 4.0), alkaline (pH 10.0), and high-temperature (60 °C) environments.

**Figure 6:**
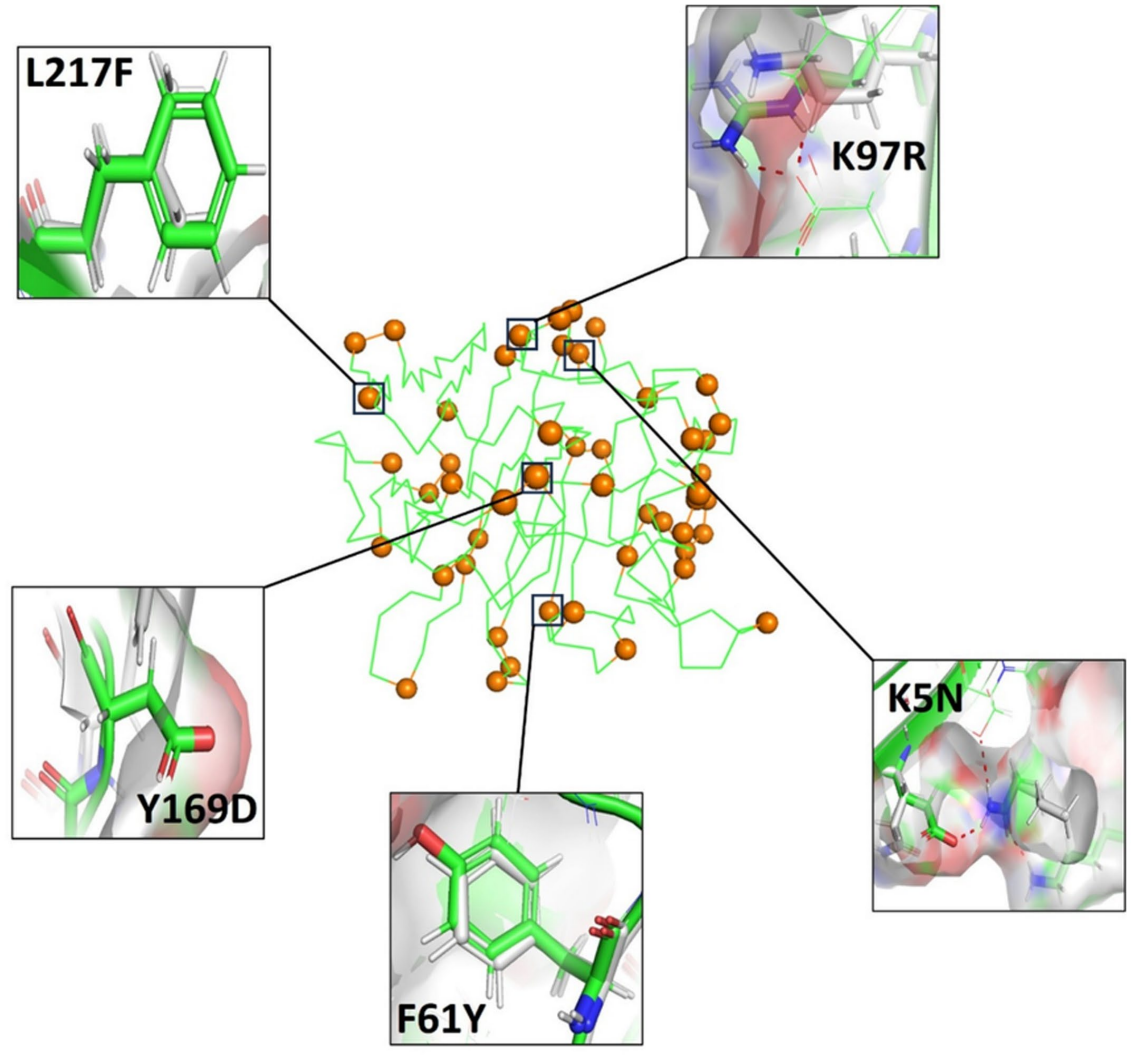
Structural details of the inferred ancestral variant AncAlyA2. Wild-type FlAlyA is shown in green, and the inferred positions are distributed throughout AncAlyA2 and highlighted orange spheres. Thumbnails highlight the deduced mutations that contributed to stabilization.

It is well established that multiple rational design strategies can enhance protein stability and heterologous expression, including the introduction of hydrogen bonds, optimization of hydrophobic core packing, modulation of surface polarity and charge distribution (Pace et al., 2014; Tych et al., 2016; Lawrence et al., 2007). In this study, a total of 49 amino acid substitutions were identified in AncAlyA2, several of which are likely to contribute to its enhanced stability (Figure 6). Among these, seven substitutions were associated with improved hydrophobic core packing. For example, substitution of leucine with phenylalanine at position 217 (L217F) introduces a rigid aromatic side chain with an expanded hydrophobic surface, thereby strengthening hydrophobic interactions within the protein core and enhancing structural rigidity. Similar strategies have been widely reported to improve enzyme thermostability. For instance, Guo et al. identified a thermostable PL6 alginate lyase (AlyRm6A) from *Rhodothermus marinus* 4,252 and demonstrated that its enhanced stability resulted from reinforced hydrophobic interaction networks and increased molecular rigidity through computational design and ΔΔG-based analysis (Guo et al., 2023). In addition, 14 substitutions were predicted to introduce new hydrogen bonds, which are critical determinants of protein conformational stability. For example, substitution of Lys5 with Asn enabled the formation of a hydrogen-bonding network with Ser255 and Glu257, while replacement of Lys97 with Arg facilitated hydrogen bond formation with the carboxyl group of Glu199. Such interactions reduce the free energy of folding and thereby stabilize the protein structure (Pace et al., 2014). Previous studies have likewise shown that introducing additional hydrogen bonds via rational design can significantly enhance the thermal and pH stability of alginate lyases and other enzymes (Jang et al., 2016; Ishak et al., 2020; Wu et al., 2023). Moreover, 10 substitutions were associated with improved surface polarity and charge distribution. As shown in Figure 6, replacement of Phe61 with Tyr increased surface hydrophilicity while retaining partial hydrophobic character, thereby improving protein solubility. Similarly, substitutions such as Ser28 → Glu and Tyr169 → Asp introduced additional negative charges on the protein surface, further enhancing stability. In contrast, seven conservative substitutions (e.g., L29V and I42V) were unlikely to exert significant functional effects. Notably, 13 substitutions could not be readily explained based on structural considerations alone, suggesting that the enhanced stability and expression of AncAlyA2 likely result from synergistic and non-additive interactions among multiple mutations rather than from individual substitutions.

In addition to limited stability, low production yield and difficulties in enzyme extraction represent major obstacles to the industrial application of naturally derived alginate lyases. To date, numerous studies have reported heterologous expression of alginate lyases in various host systems (Table 2), among which *E. coli* remains the most widely used, followed by *K. phaffii* and *B. subtilis*. However, direct comparison among these systems is challenging due to differences in enzyme sources and experimental conditions. In the present study, the same alginate lyase gene (*FlAlyA* from *Flavobacterium* sp.) was heterologously expressed in *E. coli*, *B. licheniformis*, and *K. phaffii*, enabling a systematic comparison. The results demonstrated that *E. coli* exhibited the highest enzymatic activity among the three hosts. The wild-type sequence originating from *Flavobacterium* sp. may exhibit suboptimal codon usage for *K. phaffii*, as yeast systems often require codon optimization to match their tRNA pool, unlike the well-adapted *E. coli* and *Bacillus* systems. Post-translational modifications in yeast, such as glycosylation patterns, could alter enzyme stability or activity (Ge et al., 2018). Moreover, most reported enzyme activities obtained using *E. coli* were derived from shake-flask cultures, with limited studies evaluating production at the bioreactor scale. To address this gap, fed-batch fermentation was performed in a 5-L bioreactor using a combined glucose-yeast extract feeding strategy. Under optimized conditions, constant feeding of glucose and yeast extract at 5 g/L/h and 0.4 g/L/h, the recombinant strains expressing AncAlyA1 and AncAlyA2 achieved enzyme activities of 5164.8 U/mL and 4220.1 U/mL, respectively, representing increases of 62.9 and 33.1% compared with wild-type FlAlyA. Analysis of recent molecular manipulation studies on enhancing alginate lyase enzymatic properties (as summarized in Table 2) indicates that most investigations prioritize improvements in specific activity (U/mg) and per-mass enzymatic characteristics following enzyme activity modification. Consequently, results are predominantly reported in U/mg units. A minority of studies incorporating fermentation validation utilize volumetric activity units (U/mL). In this study, beyond optimizing the enzymatic properties of the mutants, we achieved significantly higher volumetric activity (U/mL) through fermentation optimization, surpassing previously reported levels.

**Table 2:** Engineering strategies for alginate lyases and microbial fermentation.

Enzyme and source	Modification strategy	Function	Expression host and fermentation process	Final activity	Reference
VsAly7A (from Vibrio sp. QY108)	Reconstructing CD	Improved thermal stability, substrate affinity, and soluble expression level	*E. coli* BL21 (DE3) Batch fermentation in a flask	1,955 U/mg	Fu et al. (2024)
Alginate lyase (Vibrio sp. QY102)	Truncated AL	Improved secretion efficiency	*E. coli* Fed-batch fermentation	1,789 U/mL	Sun et al. (2019)
AlgL3199 (from *Vibrio* sp.)	Site-directed mutagenesis	Improved thermal stability	*E. coli* BL21 (DE3) Batch fermentation in a flask	6,084 U/mg	Chen et al. (2025)
CelPL7A (from *Cellulophaga lytica*)	Site-directed mutagenesis	Improved thermal stability	*E. coli* BL21 (DE3) Fed-batch fermentation	1,576 U/mg	Yuan et al. (2025)
Aly01 (*Vibrio natriegens* SK 42.001)	Promoter screening	Improved soluble expression level	*Bacillus subtilis (B. subtilis)* Fed-batch fermentation	99.89 U/mL	Zhou et al. (2024)
rSAGL (*Flavobacterium* sp. H63)	Codon optimization	Improved thermal stability	*K. phaffii* Fed-batch fermentation	4,044 U/mg	Li et al. (2018)
Payn ALyase (*Paenibacillus* sp. YN15)	Site-directed mutagenesis	Improved thermal stability	*E. coli* BL21 (DE3) Batch fermentation in a flask	90.3 U/mg	Zhang et al. (2023)
FlAlyA (from *Flavobacterium* sp.)	Ancestral sequence reconstruction	Enhanced thermal stability, pH stability, and expression level.	*E. coli* BL21 (DE3) Fed-batch fermentation	5,164 U/mL	This study

As demonstrated above, different glucose feeding strategies exerted pronounced effects on cell growth, substrate utilization, and enzyme production during fed-batch fermentation. DO-stat–controlled feeding promoted rapid biomass accumulation and resulted in the highest glucose consumption (177.6 g/L) and elevated specific growth rates between 9 and 21 h (Supplementary Figure S2). However, despite enhanced biomass formation, the final enzyme activity achieved under DO-stat control was lower than that obtained using 5 g/L/h constant-rate glucose feeding. This phenomenon can be attributed to the metabolic characteristics of *E. coli*. Microorganisms exhibiting Crabtree-like behavior tend to exceed their respiratory capacity at high growth rates, leading to overflow metabolism and accumulation of by-products such as acetate, which ultimately reduce protein productivity (Habegger et al., 2018). This metabolic burden likely accounts for the reduced enzyme yield observed under DO-stat feeding. In contrast, constant glucose feeding at 5 g/L/h effectively maintained glucose at near-zero levels, minimized overflow metabolism, and favored efficient enzyme synthesis. Nevertheless, excessively low glucose availability may induce nutrient limitation and impair protein expression. To overcome this limitation, yeast extract was introduced as a supplementary nitrogen source and growth promoter. Yeast extract has been reported to stimulate *E. coli* growth and enhance acetate reassimilation (Suárez et al., 1998; Nancib et al., 1991). In this study, the combined feeding strategy successfully balanced cellular growth and metabolic burden, thereby enabling both high cell density and elevated enzyme production. Overall, these results demonstrate that rational regulation of carbon and nitrogen supply is critical for maximizing alginate lyase production. The optimized fed-batch strategy developed in this study provides a robust and scalable platform for industrial-scale production of recombinant alginate lyase.

## Data Availability

The datasets presented in this study can be found in online repositories. The names of the repository/repositories and accession number(s) can be found in the article/Supplementary material.
