# Supplementary material for: Inferring an ancestral alginate lyase for improved stability and high-level expression using fed-batch fermentation
Source: Front Microbiol. 2026 Mar 17;17:1799015. doi: 10.3389/fmicb.2026.1799015 (PMC13036166; doi:10.3389/fmicb.2026.1799015)
Supplement: Supplementary file 1 [file Data_Sheet_1.pdf]

**Table S1** Primers used for PCR in this study. The underlines indicate an overlap region for splicing overlap extension PCR (SOE- PCR); Generated restriction site in bold.

| Primer name | Sequence of primer (5' to 3')[a] |
|---|---|
| **Primers for construction** | |
| *Ecoli-F* | actttaagaaggagactcgaatgcgtaacctgcagcgag |
| *Ecoli-R* | tggtggtggtgtatactcgaagtaaaacagctaagatcgactggtcc |
| *Ecoli-F-1* | actttaagaaggagactcgaaaaaaaaccgcgaacattgatctgag |
| *Ecoli-R-1* | tggtggtggtgtatactcgaatggctcacttccaggctataaat |
| *Ecoli-F-2* | actttaagaaggagactcgaaaaaaaaccgcgaacattgatctgag |
| *Ecoli-R-2* | tggtggtggtgtatactcgaatggctcacttccaggctataaat |
| *Ecoli-F-3* | actttaagaaggagactcgatataaactccctaacatcgatttgagtcattgg |
| *Ecoli-R-3* | tggtggtggtgtatactcgagtgtgacacctccagtgaataatatttgact |
| *Ecoli-F-4* | actttaagaaggagactcgatataaactgcccaaaatagatctcagtcactg |
| *Ecoli-R-4* | tggtggtggtgtatactcgagtgtgacacttcgagattatagtattttactttagcaa |
| *Bacil-F* | gtcctctctgctctttctagatgcgtaacctgcagcgag |
| *Bacil-R* | ttttcggcagatgcatctagagtaaaacagctaagatcgactggtcc |
| *Kphaffii-F* | gctgaagcttacgtagaatttctaagactgctaagattgattggtctcat |
| *Kphaffii-R* | cgcggccgccctagggaattatgagtaacttgcaaagagtaaatcttaaccttagc |
| **Primers for verification** | |
| T7-F | taatacgactcactataggg |
| T7-R | tagaggccccaaggggtta |
| *PHY-F* | atccatacccttacttgatca |
| *PHY-R* | cagatttcgtgatgcttgtc |
| *AOX-F* | ctgaaaaataacagttatta |
| *AOX-R* | taaactaccgcattaaag |

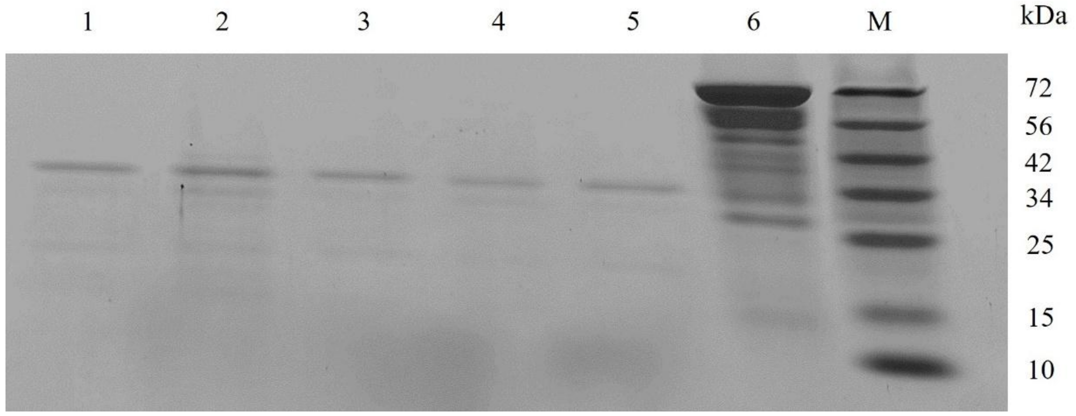


**Figure S1. The SDS-PAGE displaying FlAlyA and its infer ancestral variants.**
Line 1: FlAlyA; Line 2: AncAlyA1; Line 3: AncAlyA2; Line 4: AncAlyA3; Line 5: AncAlyA4; Line 6: 1 g/L BSA; Line M: Protein Marker.

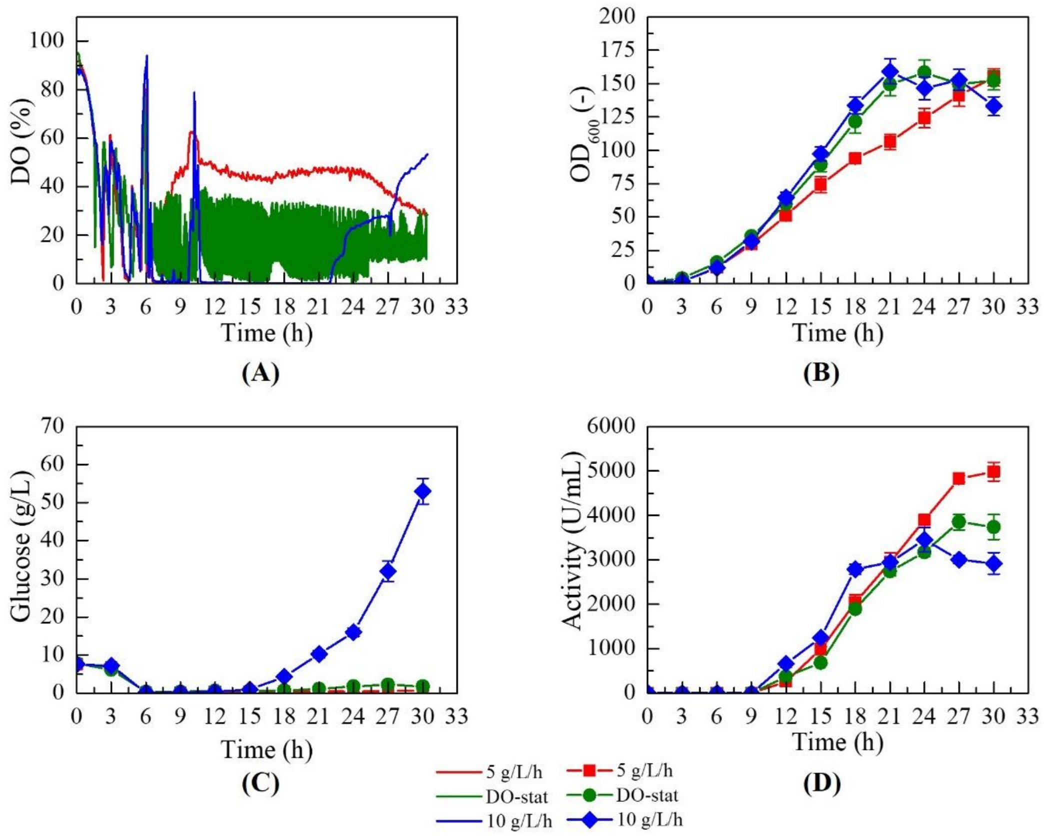


**Figure S2. Comparative analysis of fed-batch fermentation profiles using different glucose feeding strategy.**(A) DO; (B) Biomass ($OD_{600}$) ; (C) residual glucose; (D) enzyme activity.

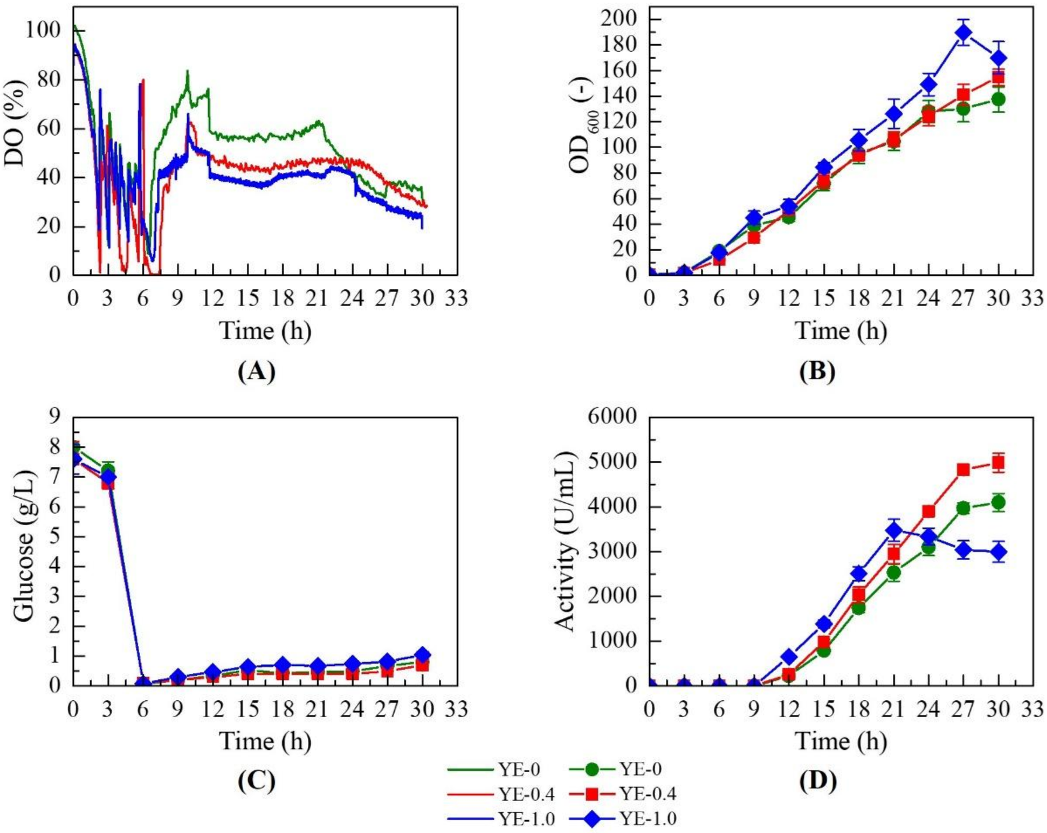


**Figure S3. Comparative analysis of fed-batch fermentation profiles using different yeast extract feeding strategy.**

(A) DO; (B) Biomass ($OD_{600}$) ; (C) residual glucose; (D) enzyme activity.

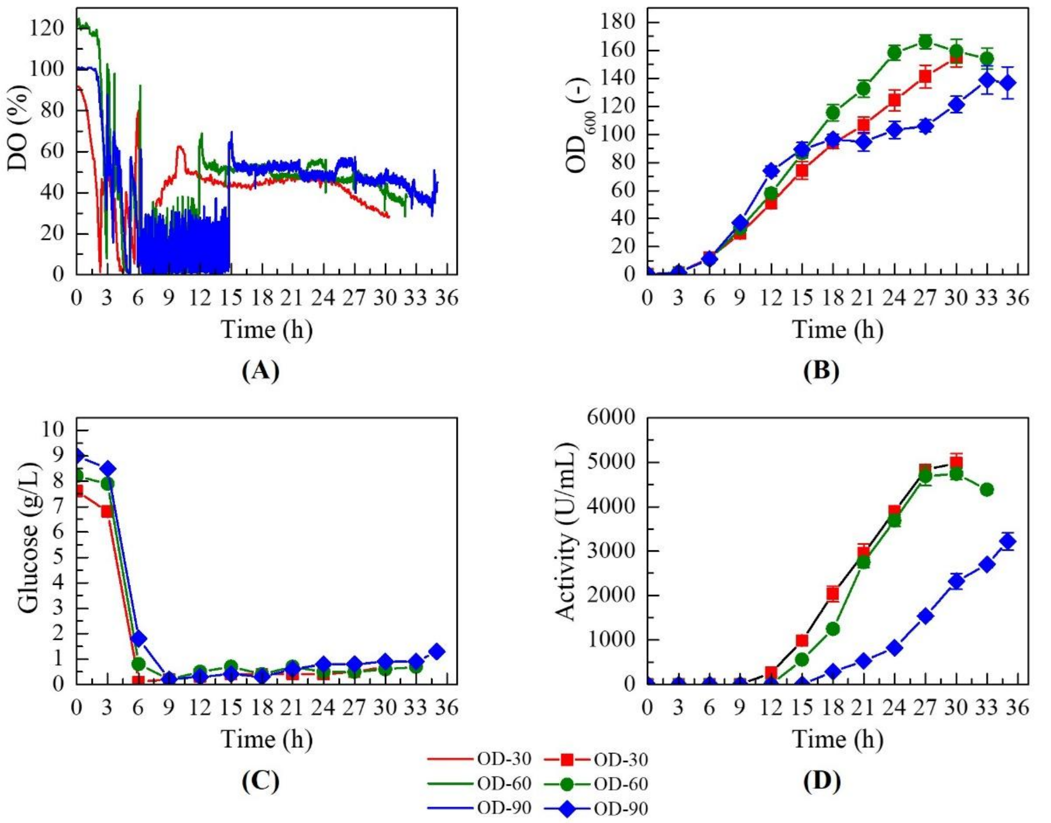


**Figure S4. Comparative analysis of fed-batch fermentation profiles using different induction $OD_{600}$.**

(A) DO; (B) Biomass ($OD_{600}$) ; (C) residual glucose; (D) enzyme activity.

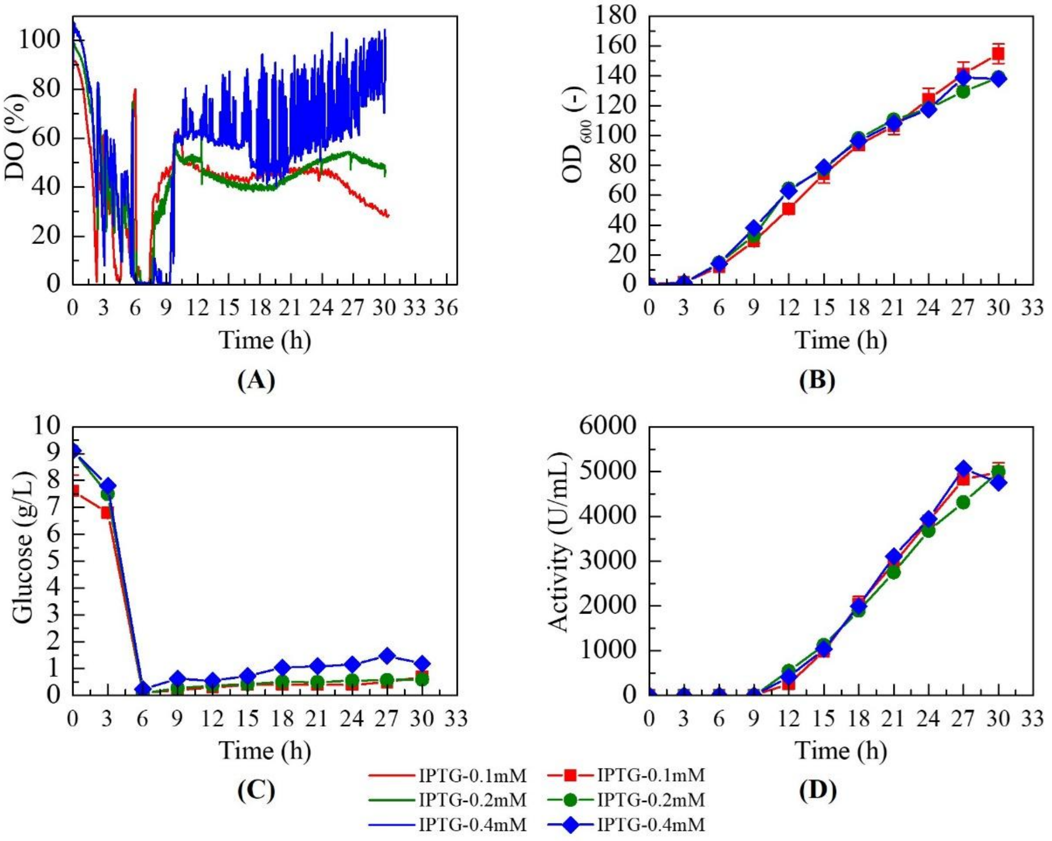


**Figure S5. Comparative analysis of fed-batch fermentation profiles using different IPTG addition.**

(A) DO; (B) Biomass ($OD_{600}$) ; (C) residual glucose; (D) enzyme activity.
